# Map2k6 is a potent genetic modifier of arterial rupture in vascular Ehlers-Danlos syndrome mice

**DOI:** 10.1172/jci.insight.187315

**Published:** 2025-01-21

**Authors:** Caitlin J. Bowen, Rebecca Sorber, Juan Francisco Calderón Giadrosic, Jefferson J. Doyle, Graham Rykiel, Zachary Burger, Xiaoyan Zhang, Wendy A. Espinoza Camejo, Nicole Anderson, Simone Sabnis, Chiara Bellini, Elena Gallo MacFarlane, Harry C. Dietz

**Affiliations:** 1 Department of Genetic Medicine and; 2 Department of Surgery, Johns Hopkins University School of Medicine, Baltimore, Maryland, USA.; 3 Wilmer Eye Institute, Johns Hopkins Hospital, Baltimore, Maryland, USA.; 4 Department of Bioengineering, Northeastern University, Boston, Massachusetts, USA.; 5 Howard Hughes Medical Institute, Chevy Chase, Maryland, USA.

**Keywords:** Cardiology, Genetics, Vascular biology, Collagens, Genetic diseases, Genetic variation

## Abstract

Aortic dissection or rupture is a major cause of mortality in vascular Ehlers-Danlos syndrome (vEDS), a connective tissue disorder caused by heterozygous mutations in the collagen type III alpha 1 chain (*COL3A1*) gene. C57BL6/J (BL6) mice carrying the *Col3a1^G938D/+^* mutation recapitulate the vEDS vascular phenotype and die suddenly of aortic rupture/dissection. However, 129S6/SvEvTac (referred to here as 129) mice expressing the same *Col3a1^G938D/+^* mutation show near-complete lifelong protection from vascular rupture. To identify genetic modifiers of vascular risk in vEDS, we performed genome-wide genotyping of intercrossed BL6/129 vEDS mice stratified by survival and identified a significant protective locus encompassing a variant in *Map2k6*, encoding mitogen-activated protein kinase kinase 6 (M2K6), a p38-activating kinase. Genetic ablation of *Map2k6* rendered previously protected 129 vEDS mice susceptible to aortic rupture, in association with reduced protein phosphatase 1 activity and increased PKC and ERK phosphorylation. Accelerated vascular rupture in vEDS mice treated with a pharmacological inhibitor of p38 was rescued by concomitant ERK antagonism, supporting an opposing role for ERK and p38 in the modification of aortic rupture risk in vEDS. These results suggest that pharmacologic strategies aimed at mimicking the effect of this natural protective pathway may attenuate aortic rupture risk in vEDS.

## Abstract 2

A genetic variant in *Map2k6* affords significant protection from death due to aortic rupture in a mouse model of vascular Ehlers Danlos syndrome.

## Introduction

Vascular Ehlers-Danlos syndrome (vEDS) is an inherited connective tissue disorder caused by heterozygous mutations in the collagen type III alpha 1 chain (*COL3A1*) gene (1–6). The clinical manifestations of vEDS include characteristic facial features, thin skin, frequent bruising, and spontaneous life-threatening arterial or hollow organ ruptures (1, 2). Disease severity and risk of vascular events can be influenced by the nature and location of *COL3A1* mutations (1–3, 7, 8). Overall, substitutions of glycine residues in the triple helical domain and splice-site mutations that lead to in-frame exon skipping are associated with a more severe phenotype than any mutational mechanism leading to functional haploinsufficiency (3). Intuitively, since type III collagen monomers interact to form a triple helical structure, seven-eighths of the total type III collagen triple helices will be abnormal if the mutant allele produces an abnormal monomer capable of homomeric interaction. In contrast, while haploinsufficiency is expected to lead to half the normal levels of type III collagen, all the resultant collagen III homotrimers will be qualitatively normal. Although these distinctions provide some insight regarding phenotypic variation in vEDS, significant clinical variability and severity of disease is observed among patients carrying mutations within a given mutational class and even between family members who harbor the identical disease alleles (6, 8–10). These observations suggest the existence of sources of genetic or environmental modification that remain to be defined (11–15).

We have previously described the generation and characterization of a mouse model of vEDS harboring a glycine substitution in the triple helical domain of type III collagen, *Col3a1*^G938D/+^, which is representative of the most common class of pathogenic variant seen in patients with vEDS (16). These mice recapitulate the vEDS phenotype, including a very high risk of spontaneous aortic rupture (16). In this study, we sought to identify genetic modifiers of vEDS by analysis of 2 mouse strains or backgrounds that have a strong influence on the rate of aortic rupture/dissection. We map the protection afforded by the 129S6/SvEvTac (referred to here as 129) background relative to the C57BL/6 (BL6) background to a single significant protective locus and identified a single gene (*Map2k6*, encoding mitogen-activated protein kinase kinase 6) as a modifier of vascular risk in vEDS, via modulation of PKC/ERK signaling.

## Results

### The 129 background protects Col3a1^G938D/+^ mice from aortic rupture.

**Figure 1:**
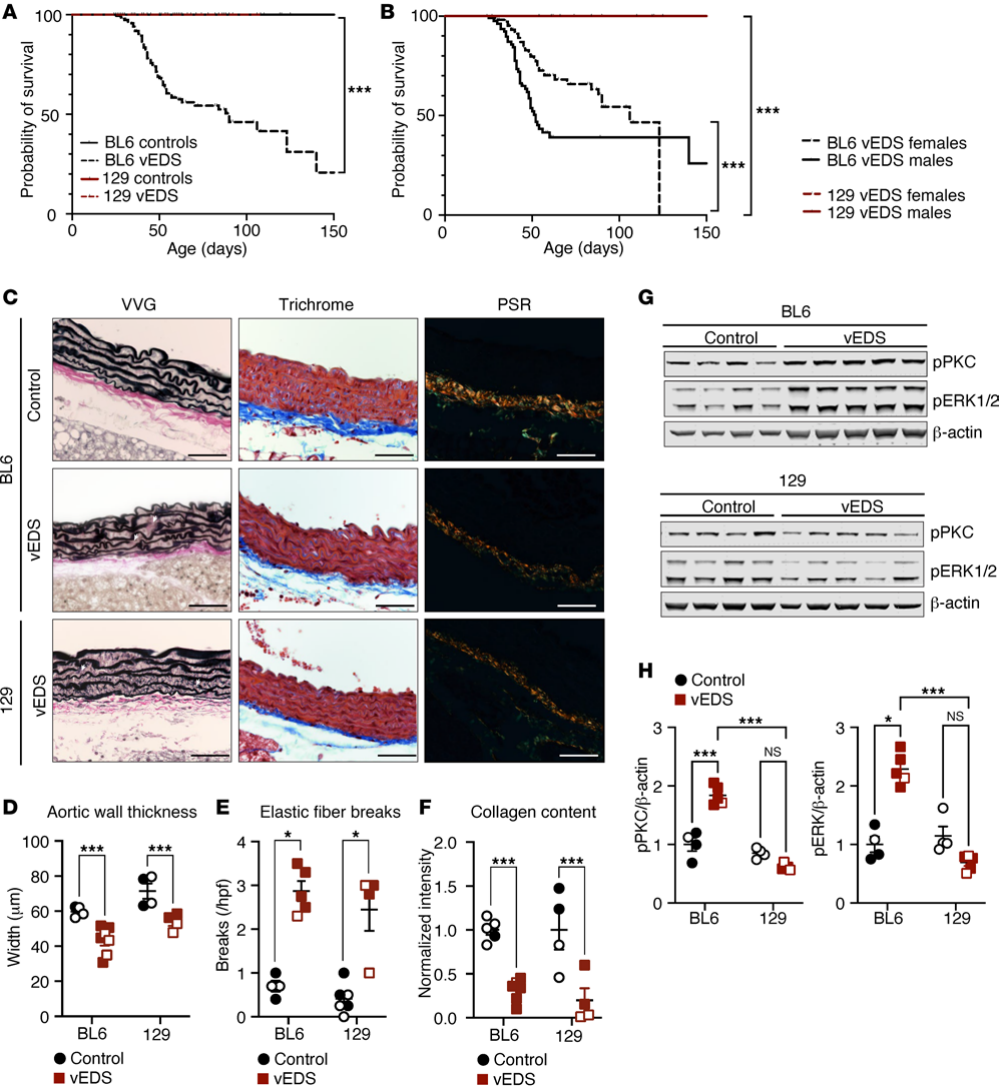
The 129Sve background protects vEDS mice from premature death. (**A**) Kaplan-Meier survival curve comparing control (*Col3a1*^+/+^) (*n* = 199) and vEDS (*Col3a1*^G938D/+^) mice (*n* = 191) on a BL6 background with control (*n* = 18) and vEDS mice (*n* = 31) on a 129 background. Significant differences were calculated using log-rank (Mantel-Cox) analysis (*** *P* < 0.001). (**B**) Kaplan-Meier survival curve comparing male BL6 vEDS male (*n* = 82) and female (*n* = 109) mice with 129 vEDS male (*n* = 15) and female (*n* = 16) mice. Significant differences were calculated using log-rank (Mantel-Cox) analysis (*** *P* < 0.001). (**C**) Verhoeff–Van Gieson (VVG), Masson’s trichrome, and Picrosirius red (PSR) staining of control and vEDS aortic cross sections at 2 months of age. White arrows indicate elastin fiber breaks. Scale bar is 50 microns. (**D**) Quantification of aortic wall thickness in aortic cross sections. *P* value refers to 2-way ANOVA with Holm-Šídák post hoc test (*** *P* < 0.001). (**E**) Quantification of elastin fiber breaks in VVG-stained aortic cross sections. Asterisks identify discovery (*q* < Q) by Kruskal-Wallis test with post hoc false discovery rate correction. (**F**) Quantification of collagen content in aortic cross sections, as measured by normalized PSR intensity. *P* value refers to 2-way ANOVA with Holm-Šídák post hoc test (*** *P* < 0.001). (**G**) Immunoblot of aortic lysates obtained from the proximal descending aortas of control and vEDS mice in the BL6 and 129 backgrounds at 2 months of age, probed with antibodies directed against phosphorylated PKCβ at residue Ser_660_ (pPKC) and phosphorylated ERK (pERK1/2). (**H**) Quantification for immunoblot shown in **G**. *P* value refers to 2-way ANOVA with Holm-Šídák post hoc test for p-PKC and Kruskal-Wallis test for p-ERK (* *P* < 0.05, *** *P* < 0.001). For **D**–**F** and **H**, each symbol represents an independent biological replicate, with unfilled symbols representing male samples. Error bars show mean ± SEM. Black circles represent control mice and red squares represent vEDS mice.

Mice of BL6 genetic background expressing the disease-causing *Col3a1* allele (*Col3a1^G938D/+^*), henceforth referred to as BL6 vEDS mice, recapitulate a severe vEDS phenotype, with spontaneous death due to aortic rupture or dissection, leading to a median survival of approximately 60 days (Figure 1A) (16). To examine if the risk of aortic rupture caused by this mutation was modified by the mouse genetic background, we backcrossed the *Col3a1^G938D/+^* mutation onto a pure 129 background. One backcross of BL6 vEDS mice to the 129 background (F_1_ generation) was sufficient to improve survival, though the effect was more modest in male compared with female mice (Supplemental Figure 1, A and B; supplemental material available online with this article; https://doi.org/10.1172/jci.insight.187315DS1). More pronounced protection from aortic rupture and premature death became apparent in both male and female vEDS mice in the F_2_ generation relative to sex-matched BL6 vEDS mice (Supplemental Figure 1, A and B), and mice with 3 backcrosses to the 129 background and above (henceforth referred to as 129 vEDS mice) showed essentially complete long-term survival in both sexes (Figure 1, A and B). This protection from vascular events on a 129 background was also recapitulated in another, less severe, vEDS mouse model (*Col3a1^G209S/+^*) (Supplemental Figure 2 and ref. 16). There were no background-specific differences in blood pressure or body mass, though both BL6 and 129 vEDS mice were slightly smaller than their wild-type littermates (Supplemental Figure 3). Alterations in aortic wall architecture in BL6 vEDS mice were relatively modest but include elastin fiber breaks, decreased aortic wall thickness, and reduced collagen content at 2 months of age (16). Each of these aortic wall abnormalities was also observed in 129 vEDS aortas, despite their improved survival (Figure 1, C–F). Analysis of passive biaxial mechanical behavior of the descending thoracic aorta of control and vEDS mice showed that increased circumferential stretch and stress in vEDS mice relative to controls was observed in the BL6 but not 129 background when evaluated at the same luminal pressure (120 mmHg), though the differences were modest and of unclear clinical significance. There were no differences observed in axial mechanical properties between vEDS mice on either background and their corresponding control group in the physiological range of loads (Supplemental Figure 4).

We have previously shown that increased activation of PKC and ERK contributes to risk of aortic rupture in vEDS mouse models (16); we thus obtained tissue from the descending thoracic aorta of 2-month-old BL6 and 129 vEDS mice, prior to any physical evidence of vascular enlargement or tear, and examined the status of these 2 signaling proteins. As previously observed, activation of ERK1/2 and PKC was accentuated in the proximal thoracic descending aorta of BL6 vEDS mice, while activation of these signaling pathways in 129 vEDS aortas was not different from that observed in wild-type littermates (Figure 1, G and H, and Supplemental Figure 5). Taken together, these data suggested the existence of protective genetic modifiers of vEDS phenotypic severity within the 129 background, possibly acting through modulation of signaling pathways previously implicated in the risk of aortic rupture/dissection in this mouse model.

### Identification of a genetic modifier using mixed background mice.

**Figure 2:**
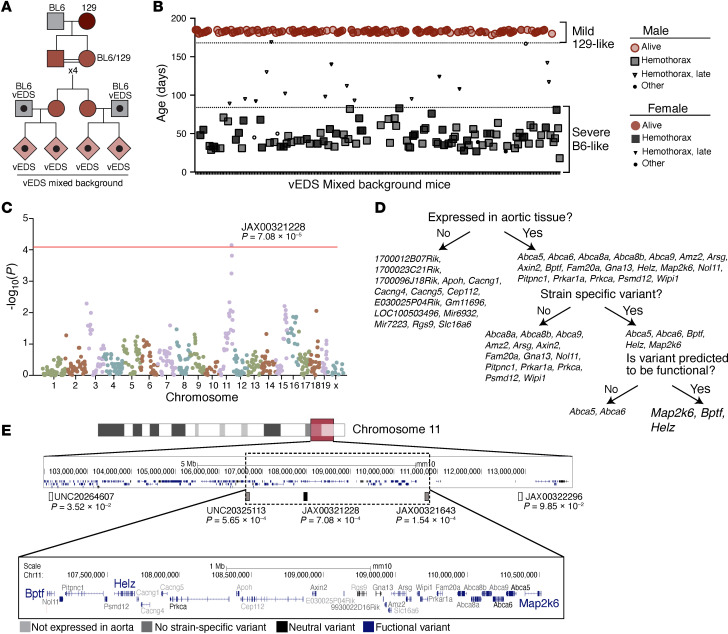
A single locus on distal chromosome 11 associates with protection from death by rupture in vEDS mice. (**A**) Breeding scheme for the generation of vEDS mice of mixed background used for GWAS. (**B**) Survival and stratification strategy of vEDS mice of mixed background. Mice that died from vascular rupture before 12 weeks of age were coded as cases (BL6-like, shown in black), while those surviving more than 24 weeks are coded as controls (129-like, shown in red). Mice that did not die from vascular rupture (i.e., malocclusion) or died between 12 and 24 weeks of age were not included in the analysis, as defined prospectively. (**C**) Manhattan plot of mixed-background vEDS mice (*n* = 91 controls, *n* = 96 cases). The red line indicates the genome-wide significance threshold. (**D**) Filtering strategy used to identify candidate modifier genes within the locus of interest. (**E**) Graphical representation of the region of interest on chromosome 11 showing annotated transcripts color-coded based on the type of variant existing in the BL6 compared with the 129 genetic background. Black and gray shapes represent BL6-like mice, and red shapes represent 129-like mice. *Bptf*, bromodomain PHD finger transcription factor; *Helz*, helicase with zinc finger.

To identify genetic variants underlying the difference in vascular rupture risk between the BL6 and 129 backgrounds, we intercrossed wild-type BL6 and 129 animals and their progeny for 4 generations (F_4_), with expansion of numbers of mice in each generation, to introduce extensive recombination between the strain-specific chromosomes (Figure 2A). Mixed-background control females were then bred to *Col3a1*^G938D/+^ male mice on a pure BL6 background to generate a large cohort of vEDS mice with a different complement of 129 alleles in heterozygosity. Introduction of the vEDS mutation was delayed until after complete intermixing and recombination to avoid any potential selection bias that could result in skewed allele distributions due to early death from aortic rupture.

This mixed-background cohort was monitored twice daily, with immediate autopsy on deceased animals to document cause of death to stratify intercrossed vEDS animals based on vascular event–related survival (Figure 2B). To maximize both signal intensity and power of downstream analyses, we focused our analysis on the extremes of survival distribution. More than 74% of all vEDS mice on the BL6 background died by 12 weeks of age, while more than 95% of vEDS mice on the 129 background lived longer than 24 weeks. Thus, death from aortic rupture between 1 and 12 weeks of age was defined as a “severe phenotype,” while survival past 24 weeks of age was defined as a “mild phenotype.” Mice that died within 1 week of birth were excluded from analysis because of ambiguity regarding the cause of death and the associated difficulty in reliable sample collection. As expected, the mixed-background population stratified into “mild” and “severe” phenotypic groups (Figure 2B). Male vEDS mice were more likely than female vEDS mice to be categorized as “severe” (*P* < 0.0001), reflective of the observed sex difference in F1 vEDS survival.

We genotyped 91 mixed-background vEDS mice with a mild phenotype (coded as controls, 62 females and 29 males) and 96 mixed-background vEDS mice with a severe phenotype (coded as cases, 29 females, 67 males). A linkage disequilibrium (LD) block-pruned set of 615 SNPs was used to perform a logistic regression GWAS using sex as a covariate. We identified 1 locus that was linked with protection from aortic rupture on chromosome 11 that attained genome-wide significance, with a peak *P* value of 7.08 × 10^–5^ (Figure 2C). The odds ratio of 0.2293 (95% CI: 0.1109–0.4741) indicated that the presence of a single 129 allele at this locus substantially decreased the odds of early death due to aortic rupture. We did not observe an association signal on chromosome X, suggesting that the increased severity in F_1_ and mixed males is not attributable to an X-linked trait but rather an autosomal trait that is sex limited or sex influenced. The study was underpowered to identify sex-specific autosomal modifiers of disease (Supplemental Figure 6).

We next leveraged genomic information and RNA transcriptomic analysis to filter genes that were present in the region of interest (Figure 2, D and E, and Supplemental Table 1). We specifically examined the 3.5 Mb region on mouse chromosome 11 that was flanked by the closest neighboring upstream and downstream SNP markers below the genome-wide significant peak on chromosome 11 (107,079,000–110,573,573 bp) (Figure 2E). We assessed this region for the presence of genes that were expressed in the aorta, and that contained putatively functional sequence variation between the 2 strains, including splice-site, nonsense and frameshift, or missense and insertion/deletion variants with a predicted impact on protein structure or function (Figure 2, D and E, and Supplemental Table 1). Of the 5 genes satisfying these criteria, *Bptf*, *Helz*, and *Map2k6* also showed differential expression in the descending aorta of mice from the 2 backgrounds based on bulk RNA-sequencing analysis (Table 1 and Supplemental Tables 1 and 2).

To narrow the scope of downstream functional analysis, we used evidence from the literature to identify, among these 3 genes, the one most likely to play a role in modulation of the vEDS phenotype. Haploinsufficiency for *Bptf* is associated with severe neurodevelopmental disability and congenital anomalies, arguing against a role in isolated modification of a vascular phenotype (17, 18). Similarly, *Helz* encodes a widely expressed RNA helicase, not suggestive of a strong relevance to vascular homeostasis (19). Based on these considerations, we thus focused downstream analyses on the missense variant in *Map2k6* (rs51129320; n.11:110490856-110490856G>A; c.G227A; p.G76E), a gene expressed in multiple aortic cell types, including smooth muscle cells and endothelial cells, and coding for mitogen-activated protein kinase kinase 6 (M2K6) (Supplemental Figure 7) (20). This glycine to glutamic acid substitution is predicted to have an effect on the function of the gene by in silico analysis (PROVEAN score –3.66).

M2K6 is 1 of the 2 upstream activators of the p38 family of kinases (p38α, p38β, p38γ, and p38δ) (21, 22). To examine if M2K6 activity was indeed altered between the protective 129 background relative to BL6, we used phospho-specific antibodies to assess phosphorylation of p38α at residues targeted by M2K6 (p-p38, Thr_180_/Tyr_182_) in the proximal descending thoracic aorta of 60-day-old mice. Immunoblot of aortas derived from 129 background mice showed increased levels of p-p38α relative to BL6 samples, independently of the genotype at the *Col3a1* locus (Supplemental Figure 8), consistent with the notion that this variant leads to intrinsically higher levels of M2K6 activity in 129 mice, which could contribute to reduced risk of aortic rupture in vEDS.

### Map2k6-dependent protection of vEDS from aortic rupture associates with increased protein phosphatase 1 activity and reduced PKC/ERK phosphorylation.

**Figure 3:**
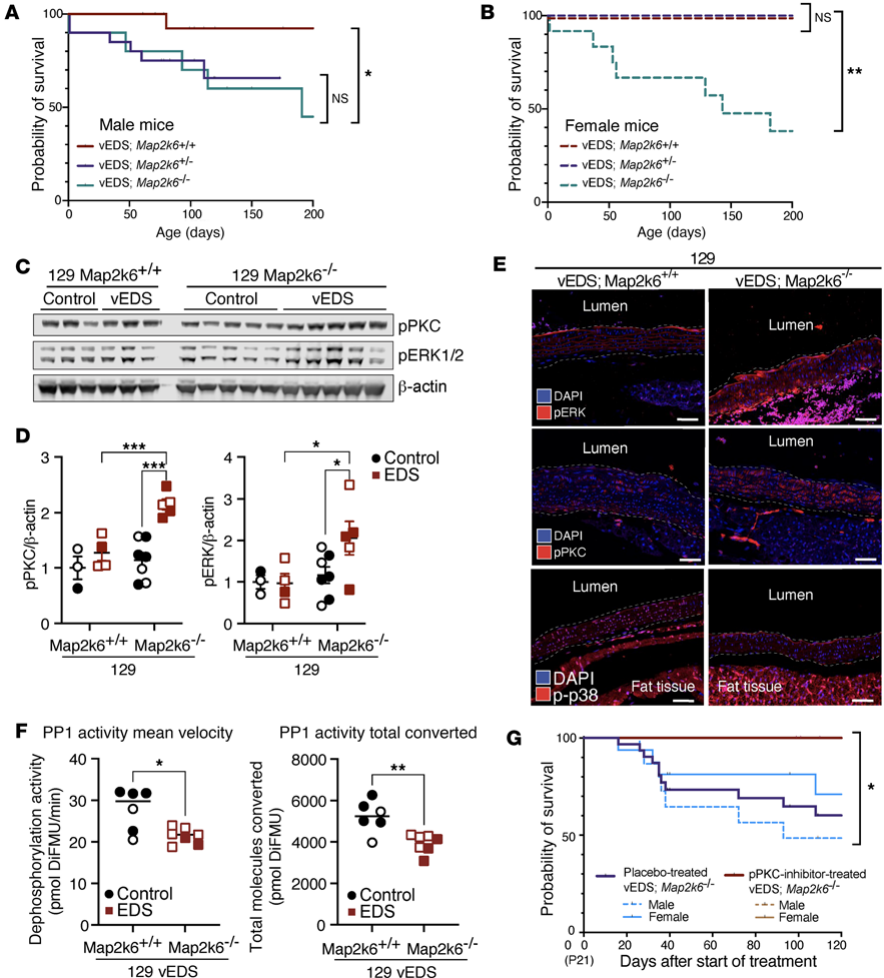
Map2k6 exerts a protective effect against death by aortic rupture in vEDS mice. Kaplan-Meier survival curve comparing (**A**) male 129 vEDS *Map2k6*^+/+^ (*n* = 17), vEDS *Map2k6*^+/–^ (*n* = 20), and vEDS *Map2k6*^–/–^ mice (*n* = 10) and (**B**) female 129 vEDS *Map2k6*^+/+^ (*n* = 19), vEDS *Map2k6*^+/–^ (*n* = 15), and vEDS *Map2k6*^–/–^ mice (*n* = 12). Significant differences were calculated using log-rank (Mantel-Cox) analysis (* *P* < 0.05; ** *P* < 0.01). (**C**) Immunoblot analysis of phosphorylated PKC at residue Ser_660_ (pPKC) and phosphorylated ERK (pERK1/2) comparing aortic lysates obtained from the proximal descending aortas of mice at 2 months of age. (**D**) Quantification of p-PKC and p-ERK normalized to β-actin of control *Map2k6*^+/+^ (*n* = 3), vEDS *Map2k6*^+/+^ (*n* = 4), control *Map2k6*^–/–^ (*n* = 7), and vEDS *Map2k6*^–/–^ (*n* = 5) mice. *P* value refers to 2-way ANOVA with Holm-Šídák post hoc test (* *P* < 0.05, *** *P* < 0.001). (**E**) Immunofluorescence of sections from the proximal descending thoracic aorta of vEDS *Map2k6*^+/+^ and vEDS *Map2k6*^–/–^ mice. The dashed line marks the approximate boundaries of the aortic wall. Scale bar is 50 microns. (**F**) Mean and total protein phosphatase 1 (PP1) dephosphorylation activity in aortic protein lysates from 129 vEDS *Map2k6*^+/+^ mice (*n* = 6) and 129 vEDS *Map2k6*^–/–^ (*n* = 7). *P* value refers to unpaired *t* test with Welch’s correction (* *P* < 0.05, ** *P* < 0.01).DiFMU, 6,8-difluoro-7-hydroxy-4-methylcoumarin. In **D** and **F**, each symbol represents an independent biological replicate, with unfilled symbols representing male samples. Error bars show mean ± SEM. (**G**) Kaplan-Meier survival curve comparing control 129 vEDS *Map2k6*^–/–^ mice (*n* = 33, 17 females and 16 males) with 129 vEDS *Map2k6*^–/–^ (*n* = 11, 8 females and 3 males) mice receiving ruboxistaurin (PKC inhibitor) starting at postnatal day 21. Significant differences were calculated using log-rank (Mantel-Cox) analysis (* *P* < 0.05).

To directly examine the role of *Map2k6/M2K6* in modulating the aortic rupture risk in vEDS, we next crossed protected 129 vEDS mice to *Map2k6*^–*/*–^ mice, also on a 129 background, to generate 129 vEDS mice haploinsufficient or fully deficient for *Map2k6*. While *Map2k6*^–*/*–^ mice have no known vascular phenotype, they have been reported to develop cardiac hypertrophy after 6 months of age in the BL6 background (23). While biallelic loss of *Map2k6* alone did not affect survival of control 129 mice (Supplemental Figure 9), it rendered previously protected 129 vEDS mice of either sex vulnerable to vascular rupture (Figure 3, A and B).

However, the effect of *Map2k6* haploinsufficiency (*Col3a1*^G938D/+^ *Map2k6*^+/–^) was sexually dimorphic, with loss of only 1 *Map2k6* allele sufficient to cause increased death in male but not female mice, paralleling the dimorphism seen in F_1_ vEDS mice (Supplemental Figure 1, A and B). Histological defects observable in the aorta of vEDS mice of either background (Figure 1) were also seen in the aorta of *Map2k6*-deficient vEDS mice, with no significant differences driven by *Map2k6* deficiency (Supplemental Figure 10). No BL6 vEDS mice with only 1 copy of *Map2k6* on a BL6 background (BL6 *Col3a1*^G938D/+^ *Map2k6*^+/–^) were observed past postnatal day 1, suggesting that the loss of 1 *Map2k6* allele in vEDS mice on a BL6 background results in complete prenatal or perinatal lethality (Supplemental Table 3).

Analogous to what was observed in vEDS mice on the BL6 background (Figure 1G and Supplemental Figure 5), increased risk of aortic rupture in 129 *Map2k6*-deficient vEDS mice associated with increased levels of PKC and ERK phosphorylation, as assessed both by immunoblot and immunofluorescence analyses (Figure 3, C–E), as well as the expected decreased p38 phosphorylation (Figure 3) (23). M2K6-activated p38 can increase activation of PP1 and PP2A (21, 22, 24), which dephosphorylate PKC and ERK, among many other substrates (24–26). We therefore hypothesized that the protective role of M2K6 may depend, at least in part, on the activation of phosphatases that dephosphorylate PKC and ERK. Consistent with this hypothesis, the descending thoracic aorta of vulnerable 129 *Map2k6*-deficient vEDS mice showed reduced levels of PP1 activity relative to protected 129 *Map2k6^+/+^* vEDS mice (Figure 3F). Levels of protein phosphatase activity were also increased in protein lysates from the descending thoracic aorta of 129 background mice compared with BL6 background mice at 2 months of age, with PP1 contributing to approximately 70% of the overall activity regardless of genotype (Supplemental Figure 11). To demonstrate direct relevance of increased PKC and ERK activation to the increased risk of death from aortic rupture in 129 *Map2k6*-deficient vEDS mice, we treated 129 *Map2k6*-deficient vEDS mice with a PKC inhibitor, ruboxistaurin, which fully rescued the phenotype with 4 months of treatment leading to 100% survival, compared to only 50% survival in placebo-treated mice (Figure 3G).

### Inhibition of p38 activation increases vascular rupture risk in vEDS mice.

**Figure 4:**
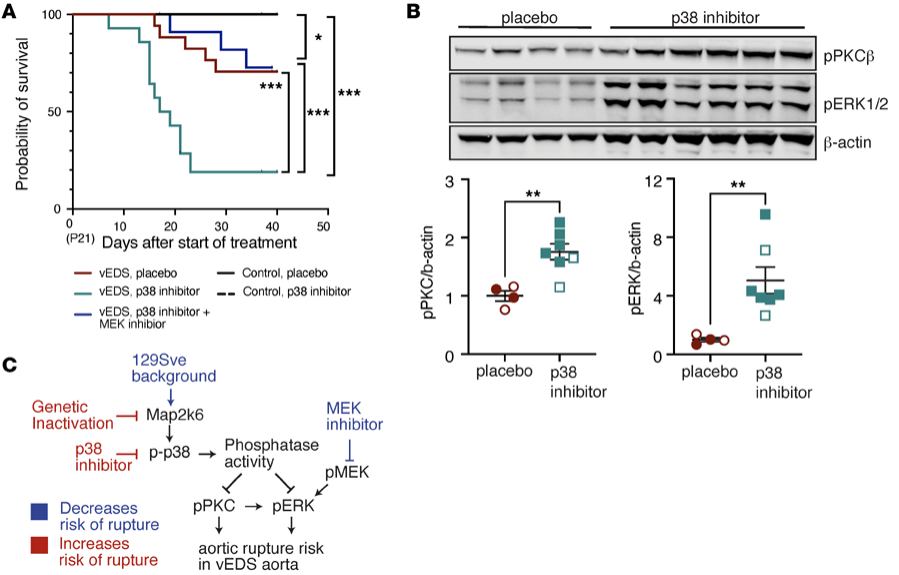
Inhibition of p38 activation increases the risk of vascular rupture in BL6 vEDS mice. (**A**) Kaplan-Meier survival curve comparing saline-injected BL6 vEDS mice (*n* = 19, 7 female and 12 male) with BL6 vEDS mice (*n* = 14, 6 females and 8 males) receiving SB203580 (p38 inhibitor) via intraperitoneal injection and SB203580-treated also receiving cobimetinib, a MEK inhibitor (*n* = 14, 6 females and 8 males), starting at postnatal day 21 and continuing for 40 days thereafter. Significant differences were calculated using log-rank (Mantel-Cox) analysis (** *P* < 0.01, *** *P* < 0.001). (**B**) Immunoblot of aortic lysates from the proximal descending thoracic aortas of BL6 vEDS mice treated with placebo or p38 inhibitor at 45 days of age, probed with antibodies directed for p-PKCβ and p-ERK and quantification of immunoblot. Each symbol represents an independent biological replicate, with unfilled symbols representing male samples. Error bars show mean ± SEM. *P* value refers to unpaired *t* test with Welch’s correction (** *P* < 0.01). Red circles represent placebo-treated mice, and blue squares represent p38 inhibitor–treated mice. (**C**) Summary figure outlining contributors to risk of vascular rupture in the aorta of vEDS mouse models.

The data presented above suggested that the M2K6/p38/PP1 pathway may play a protective role in vEDS aortic rupture risk. To directly examine the role of p38 activity on vascular rupture, we first treated BL6 vEDS mice with SB203580, a selective p38 inhibitor. Treatment significantly decreased survival in BL6 vEDS mice without affecting BL6 control mice (Figure 4A), suggesting that p38 activity has a significant protective effect on the risk of vascular rupture in vEDS mice. Surprisingly, treatment with an equivalent or doubled dose of SB203580 did not affect the risk of vascular rupture in 129 vEDS mice, though the increased dose was associated with gradual wasting, irrespective of genotype (Supplemental Figure 12). Analyses of protein lysates of the descending thoracic aorta of vEDS mice treated with the p38 inhibitor showed that the elevated risk of rupture correlated with increased levels of both PKC and ERK phosphorylation in BL6 vEDS mice (Figure 4B), while PKC and ERK phosphorylation were not affected in the 129 vEDS mice that did not demonstrate an increased risk of aortic rupture (Supplemental Figure 13). Consistent with a protective role for p38 in suppression of excessive ERK activation in vEDS aorta, elevated risk of rupture driven by inhibition of p38 in BL6 vEDS mice could be rescued by concomitant treatment with a MEK inhibitor (cobimetinib) (Figure 4A and Supplemental Figure 14). Taken together, these data suggested that the M2K6/p38/PP1 and PKC/ERK pathways play antagonistic roles in the modulation of vascular risk in vEDS mouse models (Figure 4C).

## Discussion

In this study, we show that the risk of vascular rupture is attenuated in 129 vEDS mice and that this protection associates with a protective variant in the *Map2k6* locus (rs51129320), coding for M2K6, a p38-activating kinase. Deficiency in *Map2k6* decreased the survival of otherwise-protected 129 vEDS mice, in association with reduced p38 activation, reduced PP1 activity, and increased levels of PKC and ERK phosphorylation. Similarly, pharmacological inhibition of p38 in BL6 vEDS mice resulted in increased aortic rupture risk, also in association with increased PKC and ERK phosphorylation. These observations, and the fact that increased risk of rupture due to p38 inhibition was rescued by concomitant MEK/ERK inhibition, suggest that maladaptive integration between ERK and M2K6/p38 signaling contributes to the pathogenesis of aortic rupture in vEDS animal models. The mechanisms regulating activation of these 2 pathways, and the cell types responsible for their maladaptive and adaptive functions, remain unclear.

Biallelic deletion of *Map2k6* in 129 vEDS mice did not fully recapitulate the extent of vulnerability observed in BL6 vEDS animals, suggesting the existence of additional modifying variation between the 2 strains. Furthermore, isolated pharmacologic p38 inhibition was insufficient to break the protection observed in 129 vEDS animals — even to the extent observed in 129 *Map2k6*^–*/*–^ vEDS mice. Although this may be the result of ineffective p38 inhibition, it is also possible that the protective effect of M2K6 activity may occur independently of p38 activation, for example by direct phosphorylation of members of the p21-activated kinase family (27), or that protection is imposed, and irreversible, during early development. In addition to the 129 strain, the FVB mouse strain has been shown to be resistant to β-aminopropionitrile–induced vascular rupture, and informatively this background also carries the same protective c.G227A allele at the *Map2k6* locus, suggesting a role for *Map2k6* activity in the pathogenesis of other models of aortic dissection/rupture (Ensembl database) (28).

Sexual dimorphism in survival was observed in both vEDS mice haploinsufficient for *Map2k6* and in those carrying only 1 copy of the hypomorphic BL6 variant of *Map2k6*. It remains unclear if this simply reflects the generalized enhanced vulnerability in the M2K6-deficient state, effectively unmasking added susceptibility imposed by male sex, or direct crosstalk between M2K6 and androgen signaling cascades. It is notable that activation of p38 has previously been implicated in the negative regulation of androgen receptor activity (29). Integration of androgen signaling and M2K6/p38 and PKC/ERK activation may also differ among different aortic cell types, resulting in complex crossregulation that is not explored in this work and will require analysis of mice with cell type–specific pathway inhibition. Further work should also focus on whether people with vEDS are more or less susceptible to aortic rupture based on their *MAP2K6* genetic profiles. Previous work that has identified genes that affect aortic dimensions may not be as directly relevant to this disease or model, which largely presents with spontaneous dissection without aneurysm (30, 31). Despite these limitations, the findings presented in this work suggest that pharmacologic strategies that can mimic the natural mechanism of protection observed in the 129 background through modulation of M2K6/p38/PP1 or PKC/ERK signaling pathways have a strong potential of reducing aortic rupture risk in vEDS. Perhaps more generally, this work challenges the view that clinical outcomes in vEDS are uniquely determined by an obligate structural deficiency of the tissues and highlight the therapeutic potential of addressing modifiable cellular signaling events to achieve and sustain vascular homeostasis.

## Methods

### Sex as a biological variable.

Our study examined male and female mice, and sex-dimorphic effects are reported.

### Study design.

The aim of this study was to find genes that modify the risk of vascular rupture in mouse models of vEDS. Utilizing mouse models carrying knockin heterozygous mutations in *Col3a1*, we conducted a GWAS of mixed-background vEDS mice. Sample size was calculated using power analysis (32) and relative risk assumptions based on survival data of F_1_ and F_2_ vEDS mice. Exclusion criteria were defined a priori: mice that died between 12 and 24 weeks of age were excluded, and mice that did not have clear hemothorax or hemoperitoneum on necropsy were excluded. The effects of the variant on intracellular signaling pathways were studied ex vivo. For all survival analyses, mice were censored only if unrelated to the outcome, such as for planned biochemical or histologic analysis or if the authors were directed to euthanize them by animal care staff, such as for malocclusion, fight wounds, or genital prolapse. For all other experiments, all animals were included in analyses. All experimental animals were randomized to experiment type and drug treatment. For each experiment, endpoints were defined prospectively as stated in the text. All studies were performed at a minimum of duplicate. Details regarding experimental replicates are described in the respective figure legends. Histologic and immunofluorescence analysis was performed in a blinded fashion.

### Mice.

Mice were maintained either on a C57BL/6J background (000664, The Jackson Laboratory) or on a 129S6/SvEvTac background (129SVE, Taconic Biosciences). Mice were considered 129S6/SvEvTac after backcrossing for a minimum of 8 generations, as this is when survival approached that of wild-type mice. F_1_ and mixed mice were generated by interbreeding pure C57BL/6J and 129S6/SvEvTac mice. To identify the genetic locus, mixed-background wild-type females were bred to *Col3a1*^G938D/+^ male mice on a pure BL6 background. Mice haploinsufficient for *Map2k6* were originally obtained from The Jackson Laboratory (008382) and backcrossed in the lab for a minimum of 10 generations to both a C57BL/6J and 129S6/SvEvTac background before crossing to backcrossed vEDS mice on their respective backgrounds. Restriction enzymes were used to detect the presence or absence of the *Col3a1* mutation. The G209S mutation leads to the loss of an AvaII cut site, and the G938D mutation leads to the gain of a BamHI cut site (AvaII R0153L; BamHI-HF R3136S, New England Biolabs). *Map2k6^–/–^* mice were genotyped according to The Jackson Laboratory protocols. All mice found dead were assessed for cause of death by necropsy, noting in particular hemothorax and hemoperitoneum. All mice were cared for under strict adherence to the Animal Care and Use Committee of the Johns Hopkins University School of Medicine.

### Histology and immunofluorescence.

Mice were euthanized by isoflurane inhalation, and the left common iliac artery was transected to allow for drainage. PBS and 4% paraformaldehyde (PFA) in PBS were flushed through the left ventricle. The heart and thoracic aorta were removed en bloc and fixed in 4% PFA overnight at 4°C. Aortas were submitted for paraffin fixation, and longitudinal sections 5 micrometers thick were mounted on glass slides and stained with HE, VVG, Masson’s trichrome, or PSR. Slides were imaged at 20× and 40× original magnification using a Nikon Eclipse E400 microscope. Collagen content was determined by polarized PSR intensity (33), and elastin breaks were counted by a researcher blinded to genotype using only VVG-stained longitudinal sections where elastin breaks were clearly visualized with 3–7 images per individual mouse, taken with the same 40× objective (16). All experiments were performed at 2 months of age unless otherwise specified.

For immunofluorescence, slides were incubated in antigen retrieval solution for 1 minute in a pressure cooker. Sections were incubated with 1% BSA for 1 hour at room temperature. Primary antibodies were diluted at 1:200 in 1% BSA and incubated overnight at 4°C. Three consecutive washes were performed prior to incubation with anti-mouse secondary antibodies conjugated to Alexa Fluor 594 (Invitrogen R37119) at 1:200 for 1 hour at room temperature. Slides were again washed 3 times prior to mounting with VECTASHIELD Hard Set Mounting Media with DAPI (Vector Laboratories H-1500). The following primary antibodies were used: anti–phosphorylated PKC (Abcam ab75837), anti–phosphorylated ERK (Cell Signaling Technology 4370), and anti–phosphorylated p38 (Cell Signaling Technology 4511). Images were acquired on a ZEISS 780-FCS confocal microscope at 20× original magnification and are presented as maximal-intensity projections. All images were collected at the same time and with the same settings. All experiments were performed at 2 months of age unless otherwise specified.

### Western blot.

Descending thoracic aortas (distal to the left subclavian branch and proximal to the diaphragm) from mice that did not die from aortic rupture and did not have any overt pathology at the time of planned sacrifice were harvested, snap-frozen in liquid nitrogen, and stored at –80°C until processing. Protein was extracted using an automatic bead homogenizer in conjunction with a Protein Extraction Kit (Full Moon Biosystems). All protein lysis buffers contained both PhosSTOP and cOmplete, Mini, EDTA-free Protease Inhibitor Cocktail (Roche). Western blotting was performed using LI-COR buffer and species-appropriate secondary antibodies conjugated to IRDye (IRDye-680RD goat anti-mouse 926-68070, Lot No. D11130-05, and IRDye-800CW goat anti-rabbit 926-32211, Lot No. D11215-03; LI-COR Biosciences), according to the manufacturer’s guidelines, and imaged and analyzed using LI-COR Odyssey, an infrared imaging system with a wide dynamic range that allows for quantification of both high- and low-abundance proteins. Signal quantification was determined as Signal = Total – (Background × Area), where Total is the sum of all individual pixel intensities in the defined region of interest. Each target band signal was normalized to the internal loading control signal. The following primary antibodies were used: anti–β-Actin (8H10D10) (Cell Signaling Technology, 3700), anti–phospho-ERK1/2 (Cell Signaling Technology, 4370), anti-PKCβ (phospho-Ser_660_) (Abcam, 75837), and anti–phospho-p38 (Cell Signaling Technology, 4511). All experiments were performed at 2 months of age unless otherwise specified. All protein amounts were normalized to β-actin as an internal loading control.

### Bulk RNA sequencing.

At 2 months of age, descending thoracic aortas were dissected as described above, flushed in PBS, and directly stored in TRIzol (Invitrogen). RNA was extracted according to manufacturer’s instructions and purified using the PureLink RNA Mini Kit (Invitrogen). Library prep was performed using TruSeq Stranded Total RNA with Ribo-Zero (Illumina). Sequencing was run on an Illumina HiSeq 2500 using standard protocols. Illumina’s CASAVA 1.8.4 was used to convert BCL files to FASTQ files. Default parameters were used. rsem-1.3.0 was used for running the alignments as well as generating gene and transcript expression levels. The data were aligned to mm10 reference genome. EBseq was used for differential expression analysis and default parameters were used (34)

### Gene expression.

Aortas were dissected as described above, flushed in PBS, and directly stored in TRIzol (Invitrogen). RNA was extracted according to the manufacturer’s instructions and purified with RNeasy mini columns (QIAGEN). cDNA was generated using TaqMan High Capacity cDNA Reverse Transcription reagents (Applied Biosystems), and quantitative PCR was performed in triplicate with TaqMan Universal PCR Master Mix (Applied Biosystems). The following TaqMan probes were used: Mm00803694_m1 (*Map2k6*) and Mm00446968_m1 (*Hprt*). Relative quantification for each transcript was obtained by normalizing against *Hprt* transcript abundance according to the formula 2^-Ct^/2^-Ct^ *^Hprt^*. All expression levels were normalized to untreated wild-type control expression levels.

### Delivery of medication.

Mice were initiated on medication at weaning and continued until the end of the trial. Cobimetinib (GDC-0973/RO551404, Active Biochem) was dissolved in drinking water and filtered to reach a final concentration of 0.02 g/L, giving an estimated dose of 2 mg/kg/d (16). Ruboxistaurin (LY333531 HCl, Selleck Chemicals) was mixed with powdered food (LabDiet) to give a concentration of 0.1 mg/g, giving an estimated dose of 8 mg/kg/d (16). SB203580 (Selleck Chemicals) was administered at a dose of 5 mg/kg/d or 10 mg/kg/d (doubled dose, as stated in manuscript) every 3 days by intraperitoneal injection, which had previously been shown to be effective in decreasing p38 activity in rodent models (35, 36). Animals receiving placebo (5% Tween in PBS) were also injected every 3 days by intraperitoneal injection. No drug treatments had an effect on the survival of wild-type mice (data not shown).

### Blood pressure analysis.

Blood pressures were measured by tail cuff plethysmography 1 week prior to completion of a study. To measure and record blood pressures, we utilized the BP-2000 Blood Pressure Analysis system. This method utilizes variations in the amount of light transmitted through the tail as the basic signal that is analyzed to determine the blood pressure and pulse rate. After the software determines the pulse rate, it inflates the occlusion cuff and records diastolic pressure when the wave form starts to decrease and systolic pressure when the waveform remains at a steady value. If either measurement is unclear to the software, it is not recorded. Mice were habituated to the system for 3 days prior to measurement collection in which 10–15 measurements were obtained and averaged.

### Mouse whole-genome analysis.

Genotyping was performed using the GigaMUGA array, which has been optimized to interrogate SNPs that allow discrimination of very closely related mouse strains, such as C57BL6/J and 129SveTac. Standard GoldenGate chemistry was applied on an iScan microarray scanner. SNPs were called using GenomeStudio version 2011.1, Genotyping Module version 1.9.4, and GenTrain Version 1.0 with Genome Build 37. A total of 143,446 SNPs were attempted per individual mouse (137,746 autosomal, 5,601 X chromosome, and 99 Y chromosome SNPs).

SNPs with less than 90% call rate were filtered as assay failures, which eliminated 3,942. SNPs were filtered as uninformative using the following criteria: if the minor allele frequency equaled 0 (removing 97,595 SNPs), if the heterozygote rate (AB Freq) was greater than 80% or equal to 0 (removing an additional 707 SNPs), or if the homozygote rate (AA Freq or BB Freq) was equal to 0 (removing an additional 6,461 SNPs). We then looked at genotype frequencies for parental strains (B6, 129) and F_1_ samples (50/50 mix of B6 and 129), and any SNP that did not have the 2 parental strains as opposite homozygotes was filtered as uninformative (AA or BB Freq = 0); any SNP that did not have F1 samples as heterozygotes was filtered as uninformative (AB Freq = 0). In total, 32,218 SNPs were released per individual. Of the 5,573,714 released SNPs, 7,917 no calls were made, indicating a missing data rate of 0.14%. PLINK software was utilized to prune SNPs to include only 1 per LD block using default values. PLINK software was used to calculate genome-wide association using logistic analysis and a covariate for sex. Genome-wide significance was determined with α = 0.05 and Bonferroni’s correction for the number of LD blocks. PLINK software was used to calculate the odds ratio and 95% CI.

### Phosphatase assay.

Descending thoracic aortas were dissected as described above, flushed in PBS, snap-frozen in liquid nitrogen, and stored at –80°C until processing. Protein was extracted using an automatic bead homogenizer in conjunction with a Protein Extraction Kit (Full Moon Biosystems). All protein lysis buffers for downstream phosphatase assay analysis contained cOmplete, Mini, EDTA-free Protease Inhibitor Cocktail. Lysed protein was subjected to a direct fluorescence-based assay for detecting serine/threonine phosphatase activity (RediPlate 96 EnzChek serine/threonine phosphatases Assay Kit, Molecular Probes) according to the manufacturer’s instructions. Briefly, appropriate buffers for either the total serine/threonine phosphatases or serine/threonine phosphatase PP1 were added to a 96-well microplate preloaded with inhibitors of phosphatases other than serine/threonine phosphatases, and with the fluorogenic serine/threonine phosphate substrate DiFMUP (6,8-difluoro-4-methyl-umbelliferyl phosphate), from which DiFMU is generated. The fluorescence emitted by converted DiFMU was measured using a fluorescence microplate reader, with excitation at 355 ± 20 nm and emission at 460 ± 12.5 nm. All experiments were performed at 2 months of age unless otherwise specified.

### Passive mechanical testing.

The descending thoracic aorta was excised from vEDS and control mice on both BL6 and 129 backgrounds at Johns Hopkins University School of Medicine and shipped overnight to Northeastern University for passive mechanical testing. Details on the experimental procedures and data analysis are reported elsewhere (37, 38). Briefly, following removal of perivascular tissues and suturing of the intercostal branches, the proximal descending thoracic aorta from below the left subclavian artery to the second pair of intercostal arteries was mounted on glass cannulas and coupled to a custom, computer-controlled mechanical testing device for the inflation/extension of murine vasculature. Upon estimation of unloaded length, outer diameter, and crossover axial stretch, aortic samples underwent pressurization cycles between 10 and 140 mmHg while axially extended at or ±5% the crossover stretch, and axial extension cycles to reach axial forces between 0 and 4 g, while pressurized at 10, 60, 100, and 140 mmHg. Luminal pressure, outer diameter, axial stretch, and axial force were recorded online through all testing protocols. The unloaded thickness was measured on a ring excised from either end of the aortic sample following testing. Experimental data were used for semi-inverse constitutive modeling to estimate the parameters of a strain energy density function that includes an isotropic Neo-Hookean term describing the ground matrix, and 4 families of anisotropic Fung-type collagenous fibers aligned in the circumferential, axial, and symmetric diagonal directions. Estimated constitutive parameters were used to predict biaxial stretch, stress, and stiffness at 120 mmHg luminal pressure and crossover axial stretch.

### Statistics.

All data points are presented for quantitative data, with an overlay of the mean with SEM. All statistical analysis was performed using GraphPad Prism 9 unless otherwise noted and are described in the figure legend. All data were tested for normality using Shapiro-Wilk normality tests or D’Agostino & Pearson test, depending on sample size. Normally distributed data was analyzed with 2-way ANOVA with Holm-Šídák multiple comparisons with α = 0.05 or unpaired 2-tailed *t* test with Welch’s correction. Kruskal-Wallis test followed by Dunn’s or Benjamini, Krieger, and Yekutieli (*q* = 0.05) multiple-comparison correction was used for samples that did not meet assumption of normality. Kaplan-Meier survival curves were compared using a log-rank (Mantel-Cox) test. For experiments conducted in parallel that used the same controls, statistical analysis was corrected for multiple comparisons using the Bonferroni method. Mice were censored if death was unrelated to the outcome, such as for planned biochemical or histologic analysis or if the authors were directed to euthanize them by animal care staff, such as for malocclusion, fight wounds, or genital prolapse, or if cause of death could not be ascertained.

### Study approval.

All mouse studies were performed in accordance with the guidelines of the Animal Care and Use Committee at Johns Hopkins University, which approved all animal protocols.

### Data availability.

All data generated or analyzed during this study are included in this published article, and raw data are included in the Supporting Data Values file. DNA-genotyping and RNA-sequencing data are available from the NCBI Sequence Read Archive accession PRJNA532935 and the NCBI Gene Expression Omnibus accession GSE277421. An XLS Supporting Data Values file is provided, which includes data values for all graphs and values behind any reported means in the manuscript or supplement.

## Author contributions

CJB, JFCG, JJD, and HCD contributed to the conceptualization or design of the study. CJB, ZB, GR, XZ, JFCG, RS, WAEC, NA, SS, and CB contributed to methodology and data acquisition. CJB, RS, ZB, EGM, and HCD contributed to the analysis and interpretation. CJB, EGM, and HCD supervised the project. All authors contributed to the article and approved the submitted version.

## Supplementary Material

Supplemental data

Unedited blot and gel images

Supplemental table 1

Supplemental table 2

Supplemental table 3

Supporting data values

## Figures and Tables

**Table 1:**
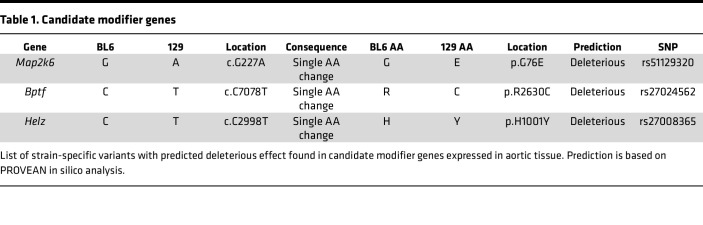
Candidate modifier genes

## Version 1 01/21/2025

In-Press Preview

## Version 2 03/10/2025

Electronic publication
