# Supplementary material for: Map2k6 is a potent genetic modifier of arterial rupture in vascular Ehlers-Danlos syndrome mice
Source: JCI Insight. 2025 Jan 21;10(5):e187315. doi: 10.1172/jci.insight.187315 (PMC11949044; doi:10.1172/jci.insight.187315)
Supplement: Supplemental data [file jciinsight-10-187315-s006.pdf]

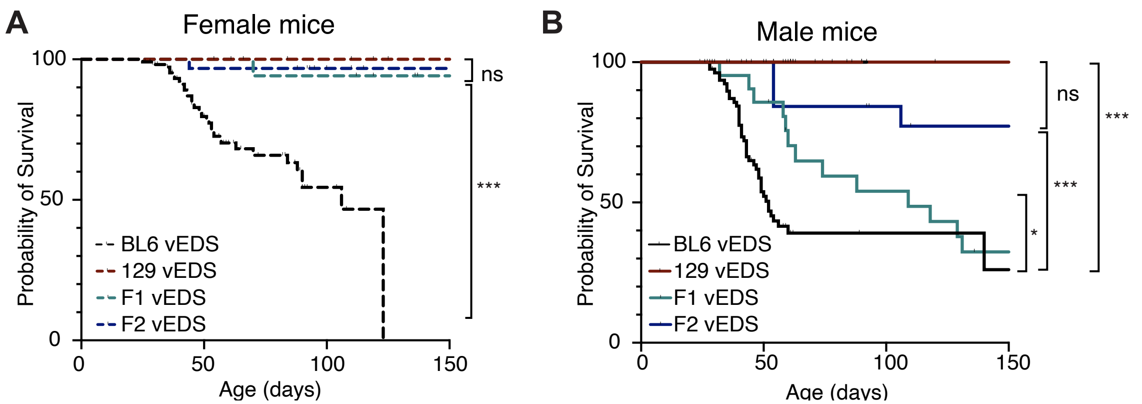

Supplemental Fig. 1. Sexual dimorphism in F1, but not F2 vEDS mice. (A) Kaplan-Meier survival curve comparing female vEDS mice after one (F1, n= 17) or two (F2, n=31) backcrosses to 129 background to vEDS female mice on pure BL6 (n=109) and 129 (n=16) backgrounds. **(B)** Kaplan-Meier survival curve comparing male vEDS mice after one (F1, n= 21) or two (F2, n=19) backcrosses to 129 background to vEDS male mice on pure BL6 (n=82) and 129 (n=15) backgrounds. Significant differences were calculated using Log-Rank (Mantel-Cox) analysis (*p<0.01, **p<0.001, **p<0.001).

Supplemental Figure 2

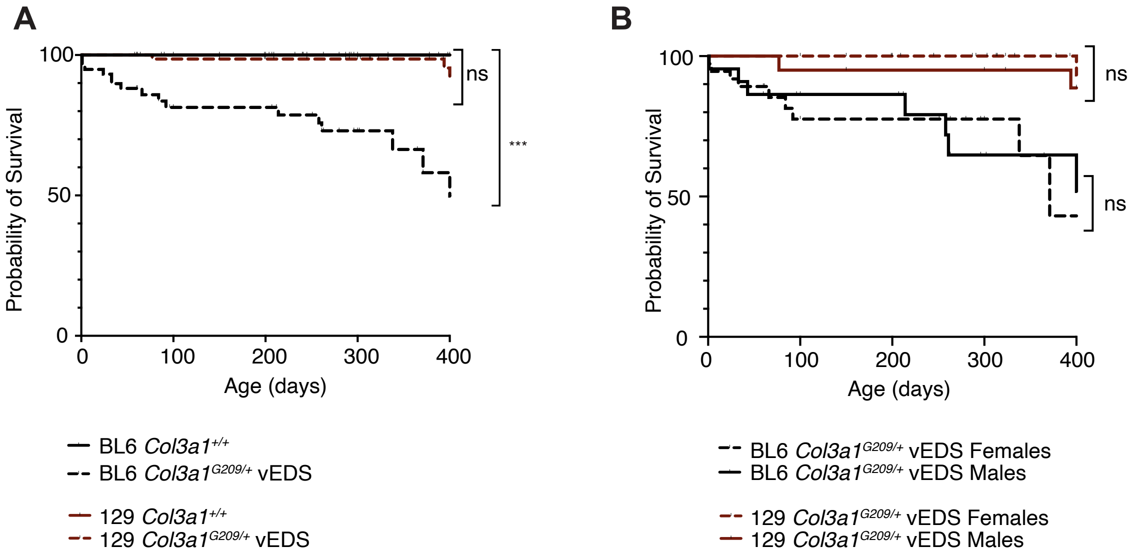

Supplemental Fig. 2. Col3a1$^{G209S/+}$ vEDS mouse model survival is also affected by background. (A) Kaplan Meier Survival curve comparing control (*Col3a1*$^{+/+}$) (n=47) and *Col3a1*$^{G209S/+}$ vEDS mice (n=59) on a BL6 background to control (n= 60) and *Col3a1*$^{G209S/+}$ vEDS mice (n=70) on a 129 background. Significant differences were calculated using Log-Rank (Mantel-Cox) analysis (*******$p < 0.001$). (**B)** Kaplan Meier Survival curve comparing male BL6 *Col3a1*$^{G209S/+}$ vEDS male (n=22) and female (n= 37) mice, and 129 *Col3a1*$^{G209S/+}$ vEDS male (n= 20) and female (n=50) mice. Significant differences were calculated using Log-Rank (Mantel-Cox) analysis.

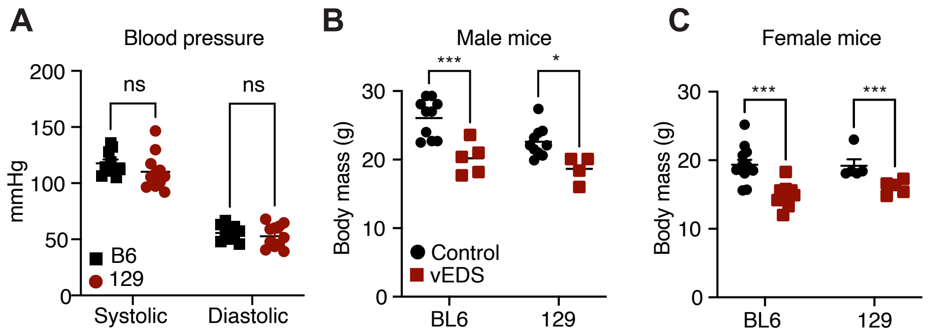

Supplemental Fig. 3. No genetic background-driven differences in blood pressure or body mass (A) Blood pressure measurements in B6 and 129 mice. **(B)** Body mass of a cohort of male vEDS mice at 60 days of age. **(C)** Body mass of a cohort of female vEDS mice at 60 days of age. Significant differences were determined using two-way ANOVA (* $p<0.05$, *** $p < 0.001$). Black symbols represent control mice and red symbols represent vEDS mice. Each symbol represents an independent biological replicate; error bars show mean ± s.e.m.

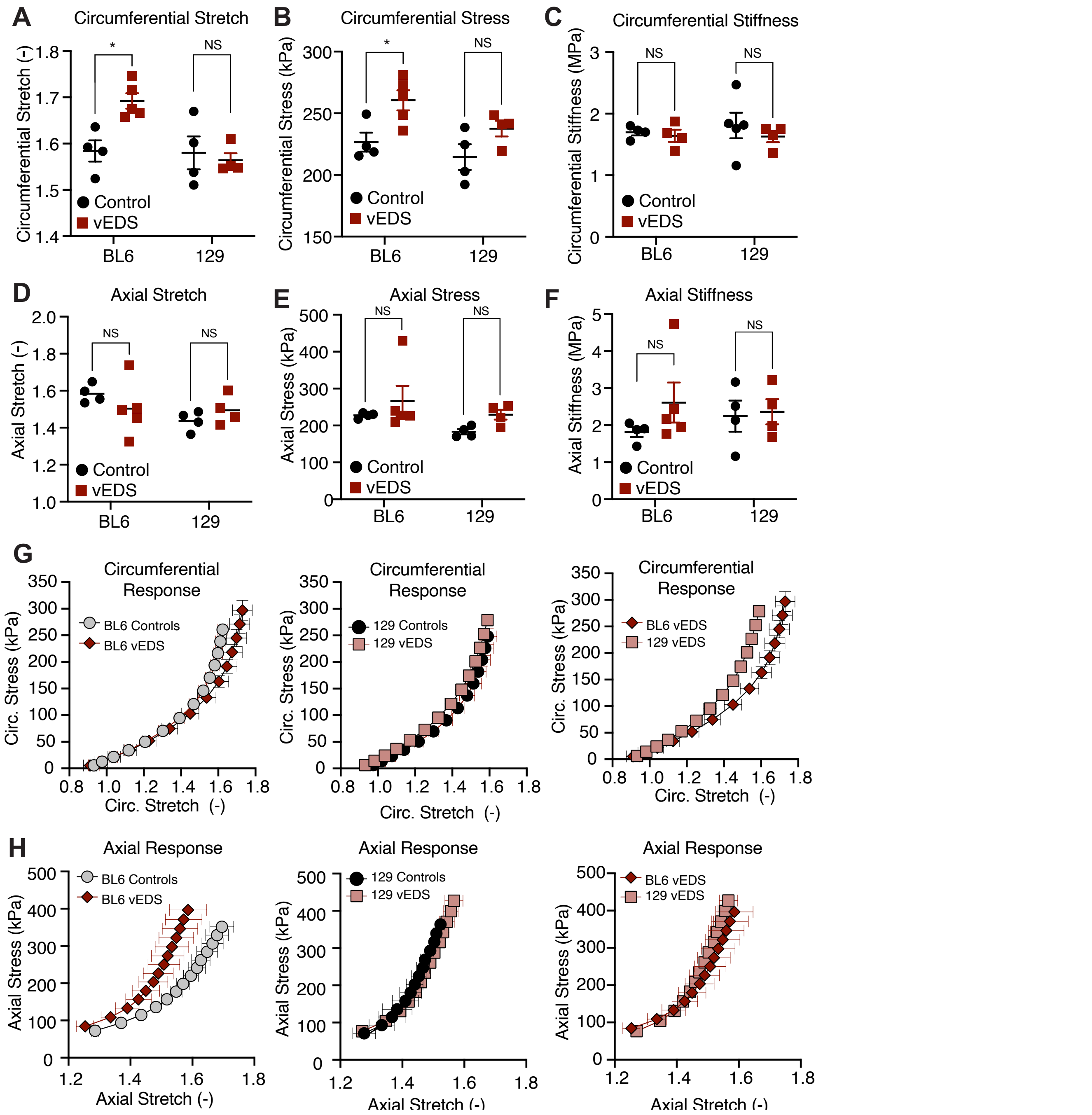

Supplemental Fig. 4. Passive mechanical properties of vEDS descending thoracic aorta in B6 and 129 background. Descriptors of descending thoracic aortic tissue mechanics in age-matched male control mice and vEDS mice of indicated genetic background at 120mmHg luminal pressure **(A)** circumferential stretch, **(B)** circumferential stress, **(C)** circumferential stiffness, **(D)** axial stretch, **(E)** axial stress, **(F)** axial stiffness. Significant differences were determined with two-way ANOVA followed by Sidak's multiple comparisons correction for normally distributed samples or Kruskal-Wallis test with Dunn's multiple comparisons test when assumptions for normal distribution were not met (*p < 0.05). Each symbol represents an independent biological replicate (n=5 for the B6 vEDS group, n=4 for all other groups); error bars show mean ± s.e.m. Average biaxial mechanical behavior of descending thoracic aortic tissues of male control and vEDS mice, illustrated by circumferential **(G)** and axial **(H)** Cauchy stress vs. stretch responses. Error bars show s.e.m. from experimental data collected on individual specimens, (n=5 for the B6 vEDS group, n=4 for all other groups). Black or grey symbols represent control mice and red symbols represent vEDS mice.

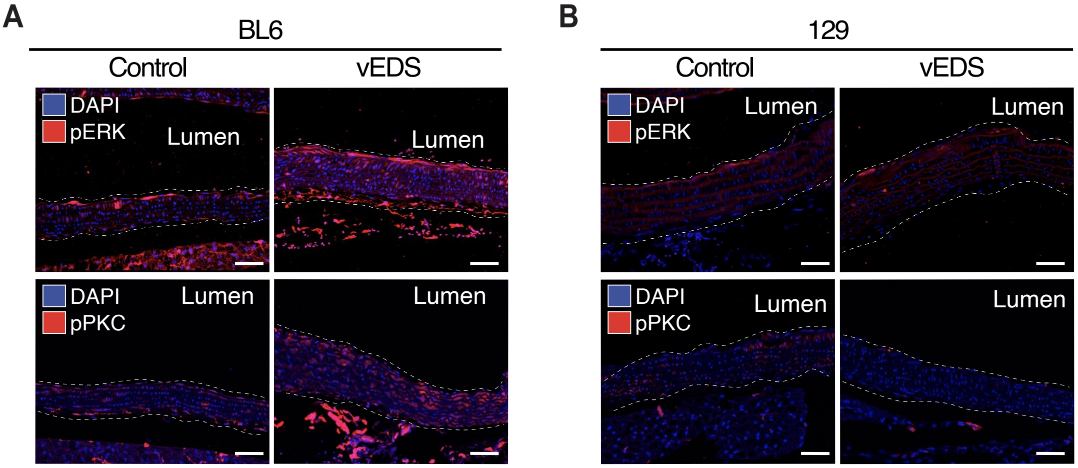

Supplemental Fig. 5. Increased levels of pERK and pPKC in the descending thoracic aorta of vEDS mice on 129 background. (A) Immunofluorescence images of sections from the proximal descending thoracic aorta of vEDS and control mice on a BL6 background probed with antibodies directed against pPKC and pERK. The dashed line marks the approximate boundaries of the aortic wall. Scale bar is 50 microns. **(B)** Immunofluorescence images of sections from the proximal descending thoracic aorta of vEDS and control mice on a 129-background probed with antibodies directed against pPKC and pERK. The dashed line marks the approximate boundaries of the aortic wall. Scale bar is 50 microns.

Supplemental Figure 6

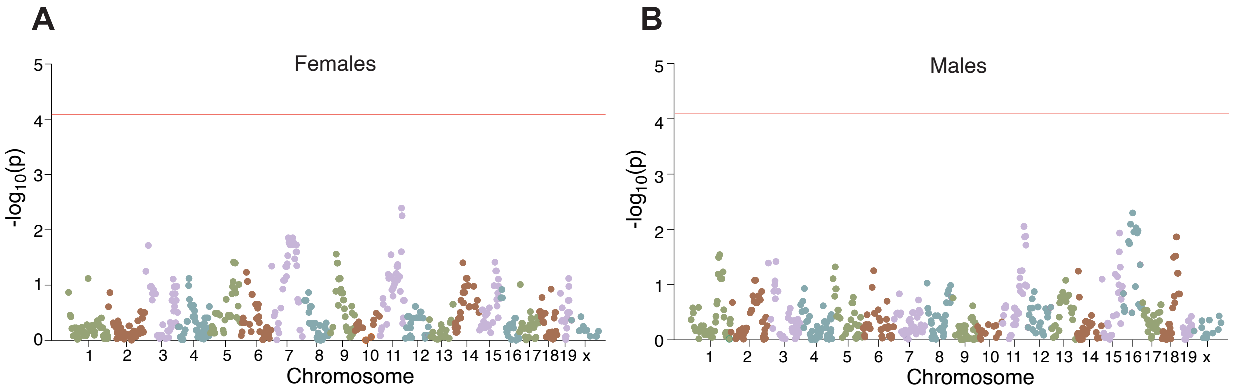

Supplemental Fig. 6. Female and male genome-wide analysis fails to identify any sex-specific locus. (A) Manhattan plot of mixed background female vEDS mice (n=62 controls and n=29 cases). Red line indicates genome-wide significance threshold. (B) Manhattan plot of mixed background male vEDS mice (n=29 controls, and=67 cases). The red line indicates the genome-wide significance threshold.

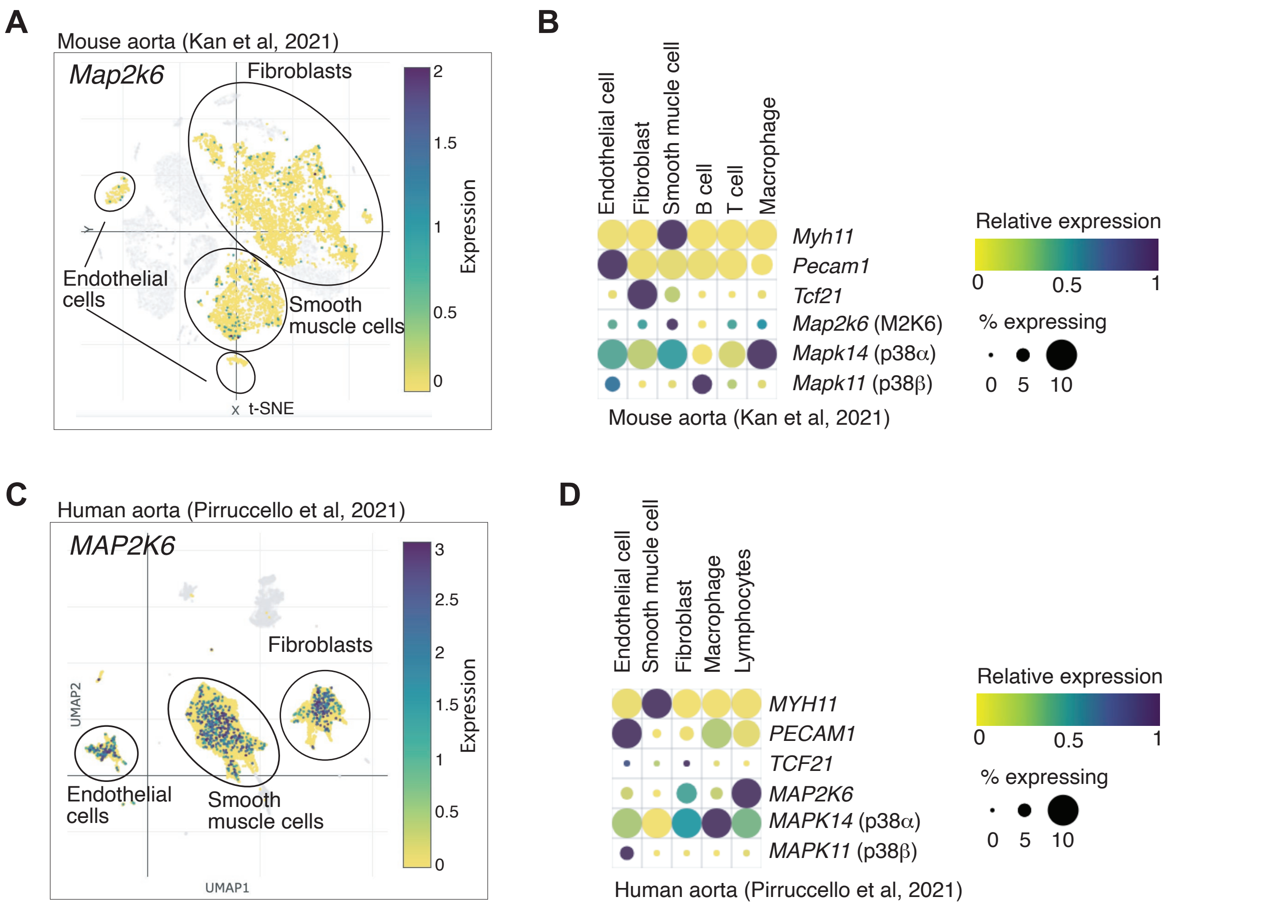

Supplemental Fig. 7. Expression of *Map2k6* and p38 kinases in aortic cell types in mouse and human aorta. (A) Cluster view of single cell RNA sequencing data of murine aorta showing expression of *Map2k6*. **(B)** Dot plot of cluster defining transcripts for fibroblast (*Tcf21*), smooth muscle cells (*Myh11*) and endothelial cells (*Pecam1*) and those coding for components of the M2K6/p38 pathway (*Map2k6*, *Mapk11*, and *Mapk14*). **(C)** Cluster view of single cell RNA sequencing data of human aorta showing expression of *MAP2K6*. **(D)** Dot plot of cluster defining transcripts for fibroblast (*TCF21*), smooth muscle cells (*MYH11*) and endothelial cells (*PECAM1*) and those coding for components of the M2K6/p38 pathway (*MAP2K6*, *MAPK14*, and *MAPK11*). All data was downloaded and analyzed via the Broad Institute Single Cell Atlas interface.

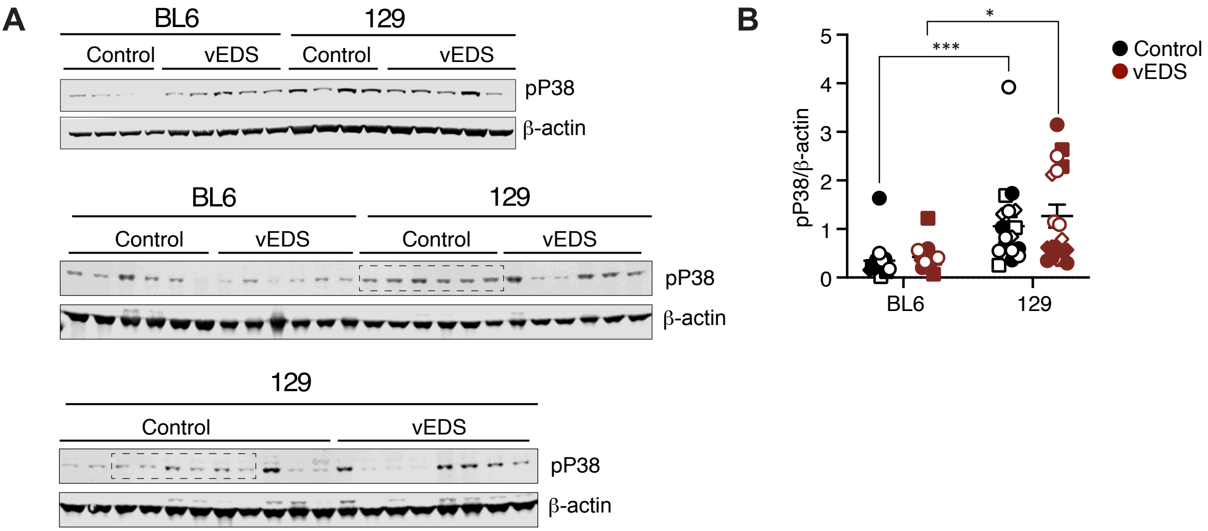

Supplemental Fig. 8. Increased p-38 phosphorylation in the aorta of mice of 129 background relative to BL6. Immunoblots of protein lysates obtained from the proximal descending aortas of control (*Col3a1*$^{+/+}$) and vEDS mice (*Col3a1*$^{G938D/+}$) in BL6 and 129 backgrounds at 2 months of age probed with antibody against phosphorylated p38 (pP38) and β-actin. Inset identifies control samples used to normalize signal across multiple immunoblots. **(B)** Quantification of pP38 levels normalized to β-actin in protein lysates from the proximal descending aorta of BL6 controls (n=15), BL6 vEDS (n=16), 129 controls (n=20) and 129 vEDS (n=17) aortas. Each symbol represents an independent biological replicate; the shape of the symbol represents an independent experiment. Black symbols represent control samples and red symbols represent vEDS samples, with clear symbols representing male samples. P-value refers to Kruskal-Wallis test with Dunn's multiple comparisons test (**** p< 0.0001, * p< 0.02) . Error bars show mean ± s.e.m.

Supplemental Figure 9

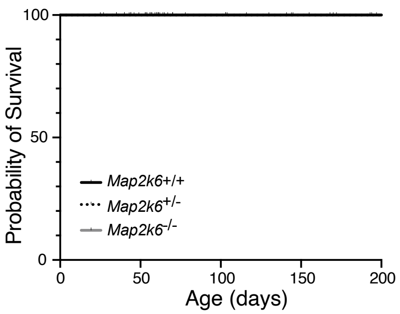

Supplemental Fig. 9. Deletion of *Map2k6* does not affect survival of control mice on the 129 genetic background. Kaplan-Meier survival curve comparing *Map2k6*$^{+/+}$ (n= 16), *Map2k6*$^{+/-}$ mice (n=111) and *Map2k6*$^{-/-}$ mice (n=71), all on a 129 background.

Supplemental Figure 10

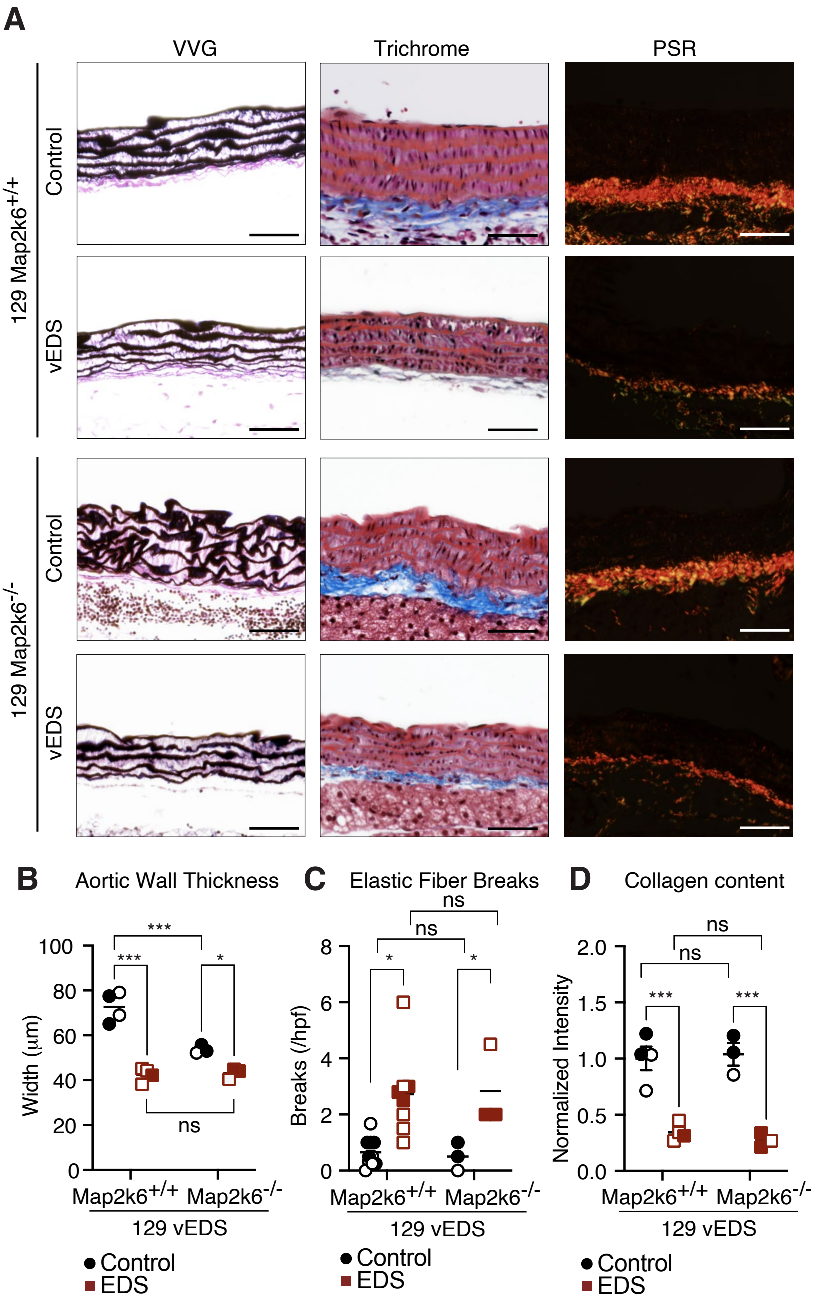

Supplemental Fig. 10. Decreased survival of *Map2k6*-deficient vEDS mice does not associate with noticeable worsening of aortic histological features relative to control vEDS mice. (A) Verhoeff Van Gieson (VVG), Masson's Trichrome, and Picrosirius Red (PSR) staining of the aorta of 2 month-old 129 control and vEDS mice, with (*Map2k6*$^{-/-}$) and without (*Map2k6*$^{+/+}$) *Map2k*6 deletion. Scale bar is 50 microns. **(B)** Quantification of aortic wall thickness in aortic cross-sections. P-value refers to two-way ANOVA with Šídák's post-hoc test (***p < 0.001). **(C)** Quantification of elastic fiber breaks in VVG-stained aortic cross-sections. Asterisks identify discovery (q < Q) by Kruskal-Wallis test with post-hoc false discovery rate correction. **(D)** Quantification of collagen content in aortic cross-sections, as measured by normalized PSR intensity. P-value refers to two-way ANOVA with Šídák's post-hoc test (*** p < 0.001). For B, C, and D each symbol represents an independent biological replicate, with clear symbols representing male samples. Error bars show mean ± s.e.m. Black circles represent control mice and red squares represent vEDS mice.

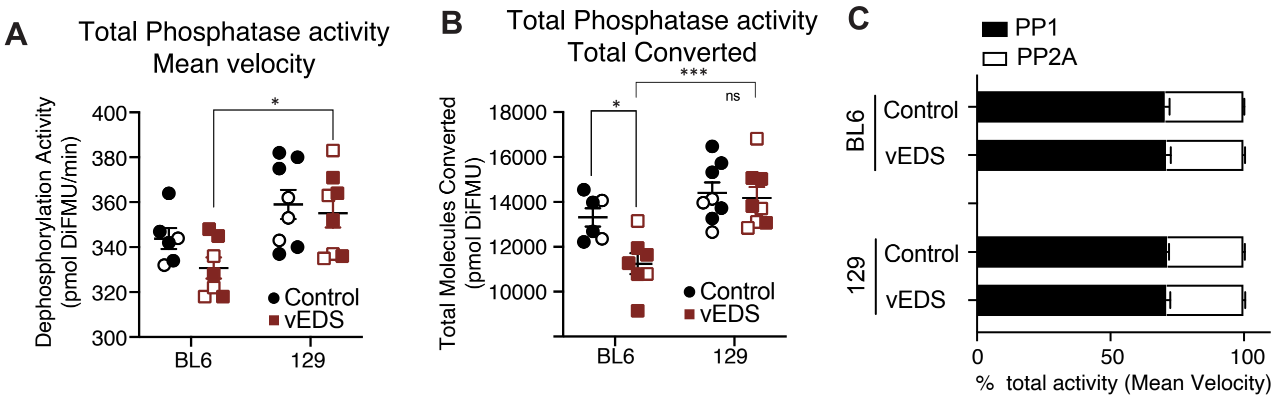

Supplemental Fig. 11. Increased phosphatase activity in the aorta of vEDS mice on the 129 background. Mean **(A)** and total **(B)** phosphatase dephosphorylation activity in protein lysates from the proximal descending thoracic aorta of control and vEDS mice on the BL6 or 129 genetic background. P-value refers to two-way ANOVA with Šídák's post-hoc test (* $p < 0.05$, *** $p < 0.001$). Each symbol represents an independent biological replicate, with clear symbols representing male samples. Black symbols represent control samples and red symbols represent vEDS samples. Error bars show mean ± s.e.m. **(C)** Proportion of PP1 and PP2A dephosphorylation activity in protein lysates from the proximal descending thoracic aorta of control and vEDS mice on the BL6 or 129 genetic background. Histogram bars represent average ± s.e.m.

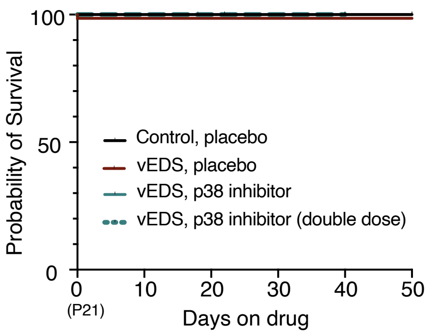

Supplemental Fig. 12. Inhibition of p38 after weaning does not affect survival of 129 vEDS mice. Kaplan-Meier survival curve comparing saline-injected 129 vEDS (n=8, 3 females and 5 males) mice, 129 vEDS (n=13, 7 females and 6 males) mice receiving SB203580 (p38 inhibitor) via intraperitoneal (IP) injection starting at postnatal day 21 and continuing for 40 days thereafter, and 129 vEDS (n=6, 4 females and 2 males) mice receiving a doubled dose of SB203580 starting at postnatal day 21 and continuing for 40 days thereafter. Significant differences were calculated using Log-Rank (Mantel-Cox) analysis.

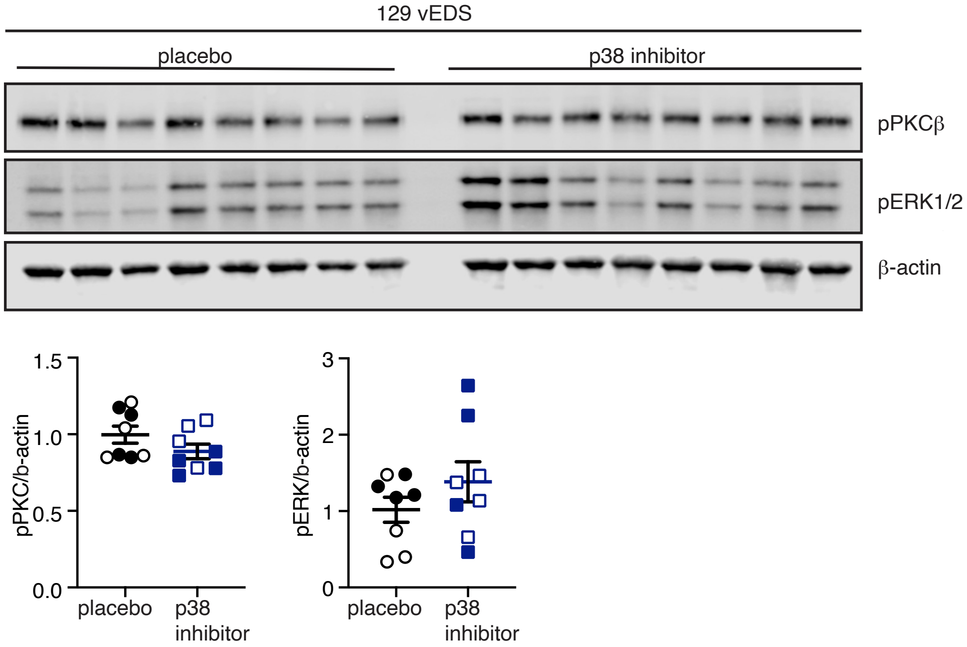

Supplemental Fig. 13. Inhibition of p38 after weaning does not affect PKC/ERK activation in 129 vEDS mice. Immunoblot of aortic lysates from the proximal descending thoracic aortas of 129 vEDS mice treated with placebo or p38 inhibitor at 45 days of age, probed with antibodies directed for pPKCβ and pERK and quantification of immunoblot. Each symbol represents an independent biological replicate, with clear symbols representing male samples. Black symbols represent control samples and blue symbols represent samples treated with a p38 inhibitor. Error bars show mean ± s.e.m.

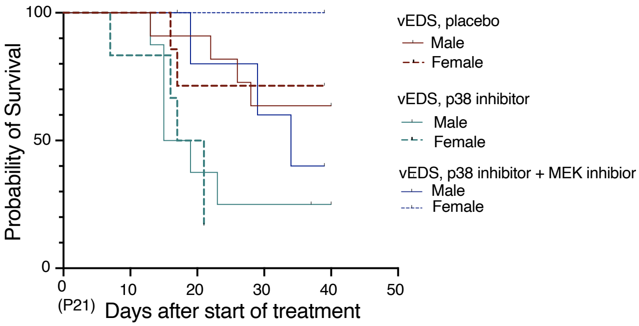

Supplemental Fig. 14. Comparison of male and female survival with inhibition of p38 activation. Kaplan-Meier survival curve comparing male and female saline-injected BL6 vEDS mice (7 female and 11 male mice) to BL6 vEDS (6 females and 8 males) mice receiving SB203580 (p38 inhibitor) via intraperitoneal (IP) injection and SB203580-treated also receiving cobimetinib, a MEK inhibitor (6 females and 8 males) starting at postnatal day 21 and continuing for 40 days thereafter.
