# Supplementary material for: Map2k6 is a potent genetic modifier of arterial rupture in vascular Ehlers-Danlos syndrome mice
Source: JCI Insight. 2025 Jan 21;10(5):e187315. doi: 10.1172/jci.insight.187315 (PMC11949044; doi:10.1172/jci.insight.187315)

Figure 1 Full Unedited Gel

Green Channel

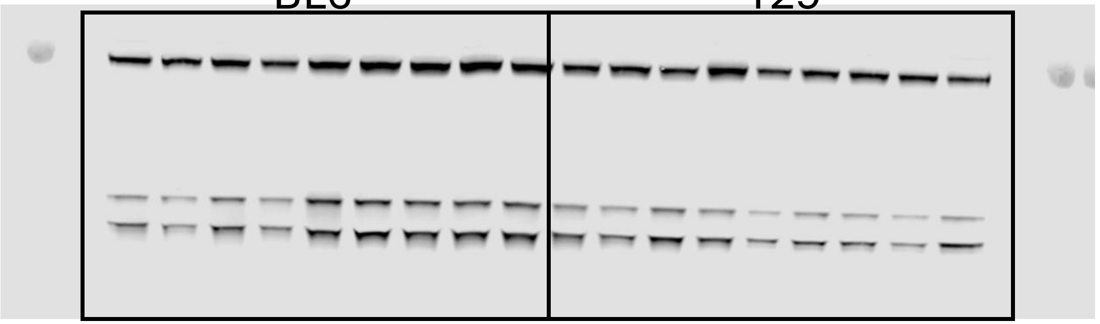

Red Channel

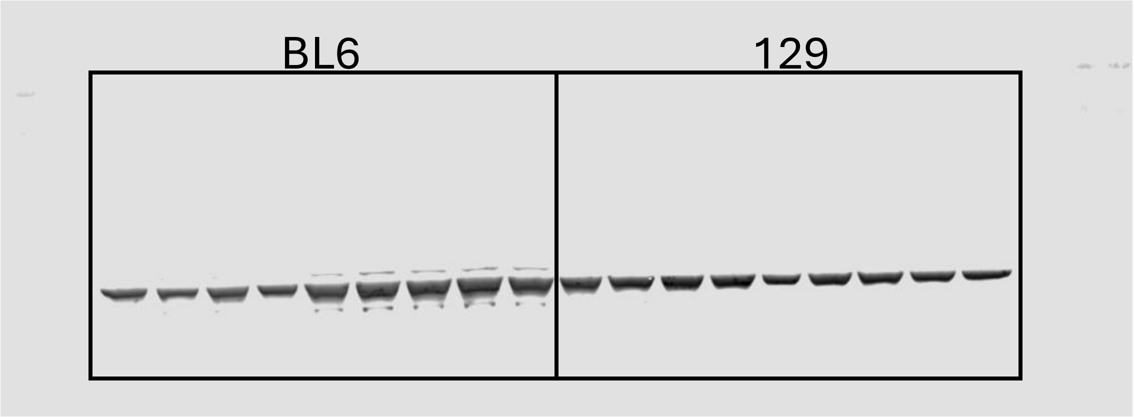

Figure 3 Full Unedited Gel

Green Channel

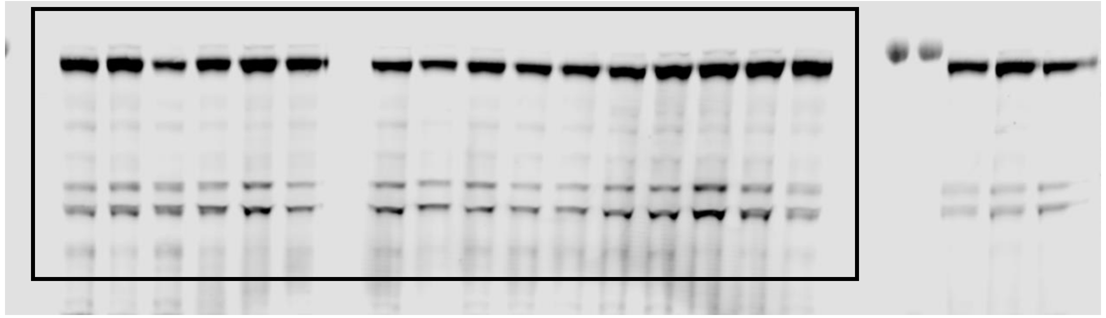

Red Channel

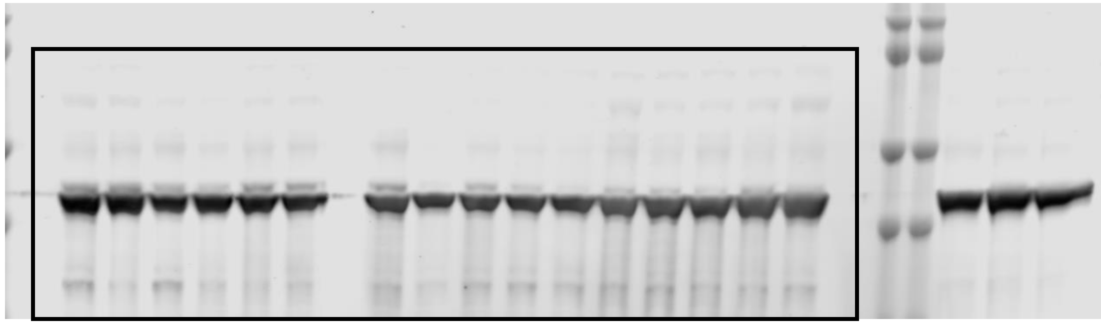

Figure 4 Full unedited gel

Green Channel

pPKC

pERK

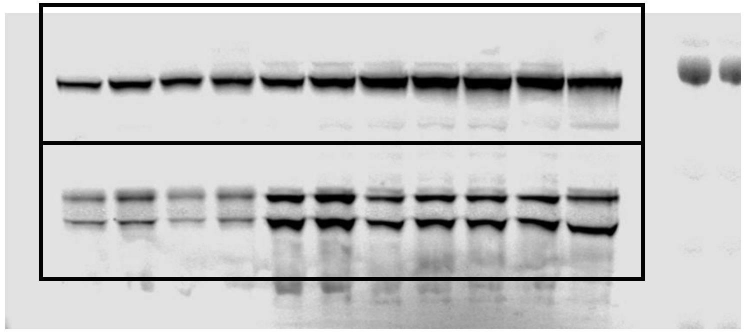

Red Channel

β actin

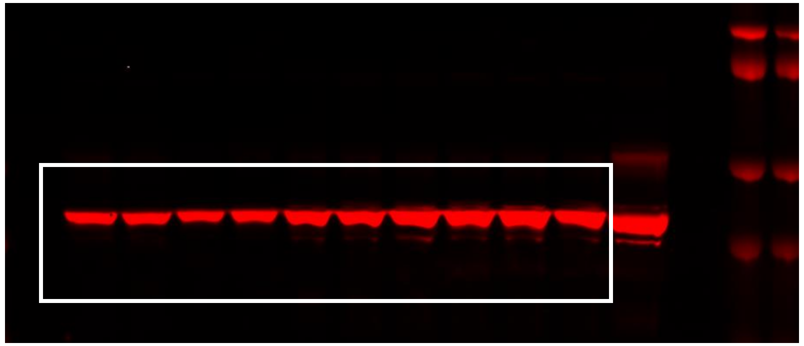

Figure Supp8 Full unedited gels

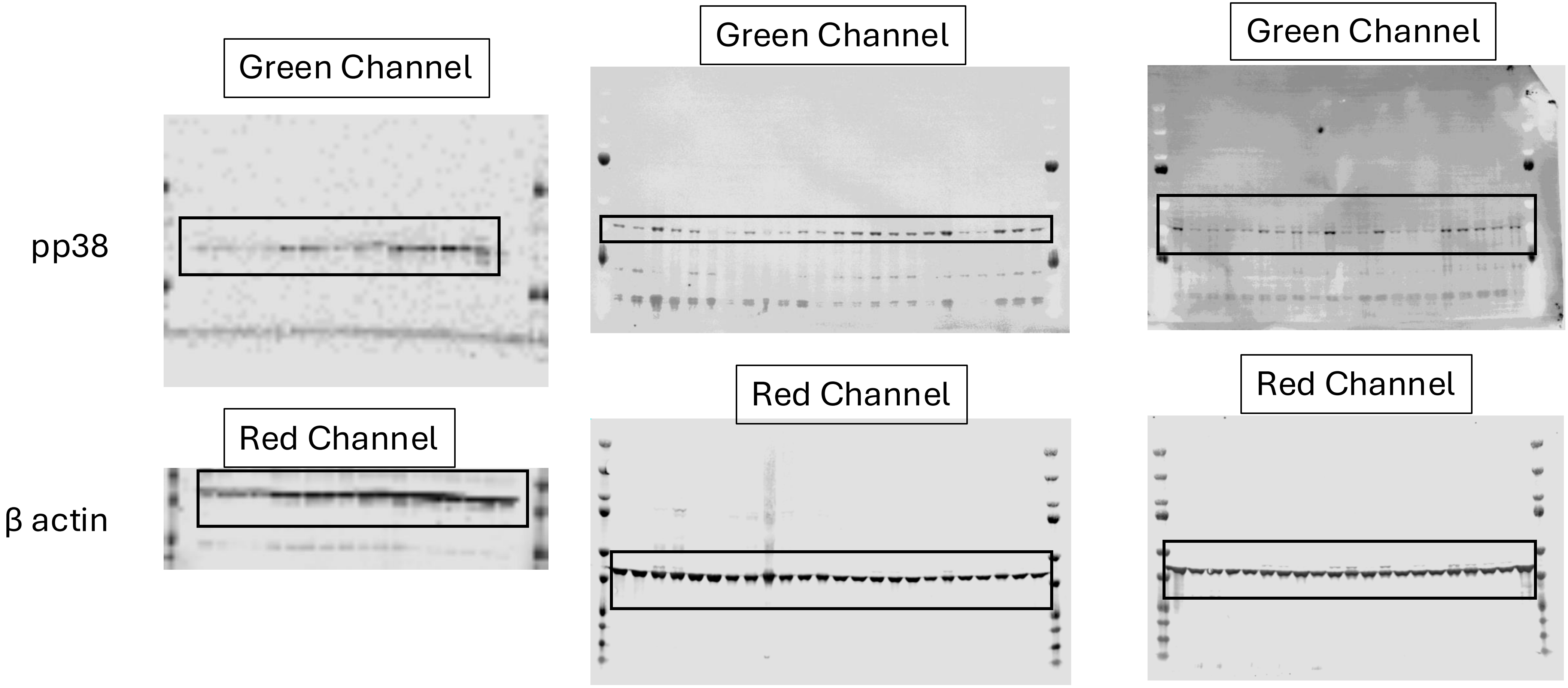

Figure Supp 13 full unedited gel

Green Channel

Red Channel

pPKC

pERK β actin

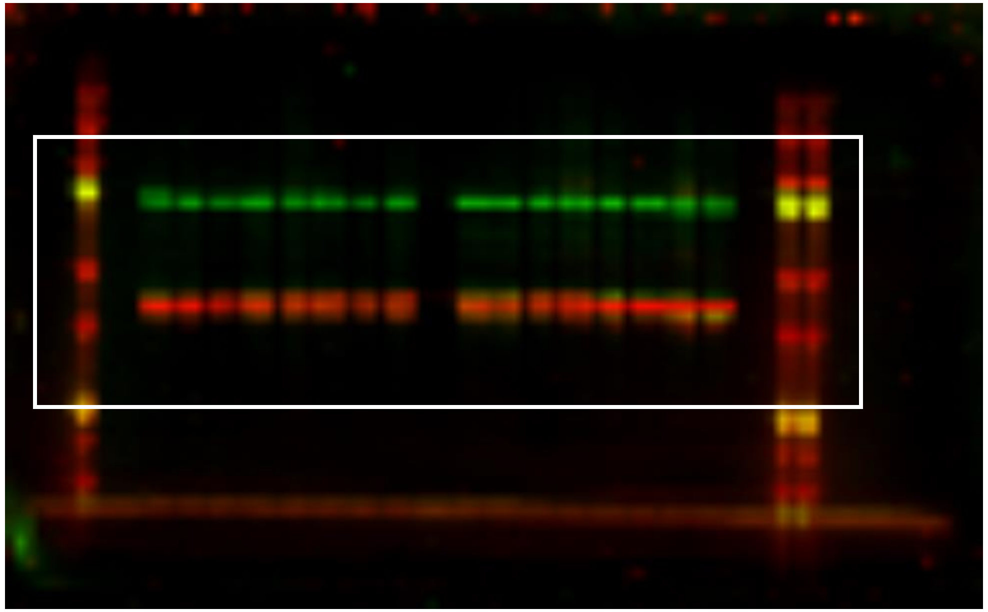

Supplement: Unedited blot and gel images [file jciinsight-10-187315-s007.pdf]
